# Genomic Profiling, Induction Response, and Transplant Outcomes in Pediatric Acute Myeloid Leukemia: A Single-Center Retrospective Cohort Study

**DOI:** 10.3390/ijms27135832

**Published:** 2026-06-28

**Authors:** Ana Maria Bicǎ, Andra Daniela Marcu, Cristina Georgiana Jercan, Iuliana Iordan, Letiția Elena Radu, Irina Avramescu, Cerasela Jardan, Dumitru Jardan, Onda Tabita Cǎlugǎru, Anda Mocanu, Andrei Colițǎ, Anca Colițǎ

**Affiliations:** 1 Faculty of Medicine, University of Medicine and Pharmacy Carol Davila, 050474 Bucharest, Romania; ana-maria.birsan@drd.umfcd.ro (A.M.B.); cristina.jercan@umfcd.ro (C.G.J.); iuliana.iordan@drd.umfcd.ro (I.I.); letitia.radu@umfcd.ro (L.E.R.); irina.avramescu@drd.umfcd.ro (I.A.); cerasela.jardan@umfcd.ro (C.J.); onda-tabita.lupu@drd.umfcd.ro (O.T.C.); anda.purnavel@drd.umfcd.ro (A.M.); andrei.colita@umfcd.ro (A.C.); anca.colita@umfcd.ro (A.C.); 2 Department of Pediatrics and Bone Marrow Transplantation Unit, Fundeni Clinical Institute, 022328 Bucharest, Romania; 3 Department of Bone Marrow Transplant, Fundeni Clinical Institute, 022328 Bucharest, Romania; 4 Department of Hematology Laboratory, Fundeni Clinical Institute, 022328 Bucharest, Romania; 5 Molecular Biology Laboratory, MedLife, 010719 Bucharest, Romania; djardan@medlife.ro; 6 Department of Hematology, Colțea Hospital, 050098 Bucharest, Romania

**Keywords:** pediatric acute myeloid leukemia, genomic profiling, risk stratification, induction response, hematopoietic stem cell transplantation, next-generation sequencing

## Abstract

Pediatric acute myeloid leukemia (AML) is biologically heterogeneous, and genomic profiling increasingly informs risk stratification and treatment. We evaluated the relationship between induction response, genomic risk, transplant allocation, and survival in pediatric AML. We retrospectively analyzed 38 pediatric patients with newly diagnosed AML, treated between 2020 and 2025. Clinical, cytogenetic, molecular, treatment, and outcome data were collected. Genomic alterations were assessed using cytogenetics, fluorescence in situ hybridization (FISH), molecular testing, and next-generation sequencing (NGS). Survival was estimated by Kaplan–Meier analysis, and prognostic factors for event-free survival (EFS) were assessed using univariable Cox regression. This study is exploratory given the limited sample size and should be interpreted accordingly. Complete remission (CR) after the first course of induction was achieved in 25/38 patients (65.8%), partial remission (PR) in 3/38 (7.9%), and refractory disease in 10/38 (26.3%). Twenty-four patients underwent allogeneic hematopoietic stem cell transplantation; 17/24 (70.8%) were alive at last follow-up, with a 2-year overall survival rate of 72.9%. Both induction response and genomic risk stratification showed suggestive associations with outcome; descriptively, induction response showed the strongest prognostic discrimination, with achievement of CR associated with markedly improved survival. High cytogenetic risk and FLT3-ITD were significantly associated with inferior EFS. Post-induction measurable residual disease (MRD) positivity was detected in 16 of 38 patients (42.1%) and was associated with suboptimal induction response; MRD negativity did not uniformly preclude adverse outcomes, particularly in the high-risk genomic subgroup. Genomic profiling refined biological risk and post-remission treatment allocation. Integrated assessment of genomic risk, induction response, and MRD status may improve therapeutic stratification in pediatric AML.

## 1. Introduction

Acute myeloid leukemia (AML) is the second most common leukemia in children after acute lymphoblastic leukemia, accounting for 20–25% of pediatric leukemia cases and an incidence of seven cases per 1,000,000 children per year [1,2]. Pediatric AML is characterized by marked biological heterogeneity, distinguishing it from adult AML and contributing to its clinical complexity [3,4]. Today it is no longer viewed as a single disease but as a group of different conditions defined by distinct genetic and molecular abnormalities [1,5]. This transformation has fundamentally reshaped clinical management, transitioning from standardized treatment approaches to precision medicine strategies tailored to each patient’s genomic profile [6,7,8].

In the last decade, advances in genomic technologies, particularly next-generation sequencing (NGS), have significantly enhanced our understanding of the molecular basis of pediatric AML [9,10]. Large-scale sequencing analyses have revealed that pediatric AML is defined by a complex spectrum of chromosomal abnormalities, gene fusions, and somatic mutations that contribute to leukemogenesis and influence the clinical course of the disease [1,11,12,13]. These studies have demonstrated that the genomic landscape is substantially different in children than in adults, with structural variants and candidate fusion events playing a prominent role in younger patients [14]. The identification of these genetic alterations has improved our understanding of the biological mechanisms driving AML development and has highlighted the heterogeneous nature of the disease.

The identification of recurrent molecular alterations has enhanced the ability to stratify patients into clinically relevant risk groups. Now we recognize the genetic alterations as key determinants of prognosis and treatment response, being able to distinguish patients with low-, intermediate-, or high-risk disease [15,16]. The genomic information is increasingly integrated into new classification systems and treatment protocols, guiding therapeutic decisions and optimizing patient outcomes [5,6,11,17]. Evidence indicates that distinct genetic abnormalities are associated with variable survival outcomes, relapse risk, and an increased sensitivity to specific therapies, underlining the important role of molecular diagnostics in the management of pediatric AML. Ongoing advances in genomic research continue to refine our understanding of this disease by identifying previously unknown molecular subtypes. Furthermore, recent genomic analyses have supported revised classification frameworks for pediatric AML based on recurrent patterns of genetic alterations and gene expression profiles, in line with the updated WHO 2022 and ICC 2022 classifications. These data indicate that pediatric AML comprises biologically and clinically distinct molecular subgroups, each defined by specific genomic features and associated clinical outcomes [14,18]. Incorporation of these molecularly defined subgroups into diagnostic classification is expected to enhance risk stratification and support the development of more precise therapeutic approaches. Genetic markers such as *FLT3-ITD*, *NPM1*, *CEBPA* mutations, and *KMT2A* rearrangements are now routinely evaluated to stratify patients into distinct groups regardless of blast percentage. Additionally, this risk-adapted approach is helping in tailoring therapy intensity, optimizing treatment outcomes, reducing treatment-related toxicity, and guiding the indication of hematopoietic stem cell transplant (HSCT) in first complete remission (CR) [19,20,21].

Despite these advances, pediatric AML is still a challenging disease; relapse continues to be a major cause of treatment failure and is largely driven by clonal evolution and disease complexity [13,22,23]. Although the survival rate has improved in recent decades, the outcome remains heterogeneous among different molecular subgroups [3,7,24].

## 2. Results

A total of 38 patients with newly diagnosed AML were included in the primary analysis (Table 1). The cohort was predominantly male (60.5%), with females accounting for 39.5%. At diagnosis, almost half of the patients were aged ≥10 years (47.4%), while 44.7% were between 1 and 9 years and 7.9% were younger than 1 year. Most patients presented with a white blood cell (WBC) count < 50 × 10^9^/L (57.9%), whereas 28.9% had hyperleukocytosis (≥100 × 10^9^/L). Based on the FAB classification, M1 and M5 were the predominant subtypes (28.9% and 31.6%, respectively), followed by M4 (26.3%), with the remaining subtypes occurring at lower frequencies.

The overall survival (OS) at 2 years of the AML cohort (*n* = 38) was 69.9%, while event-free survival (EFS) was 65.9%.

Of the 38 patients included in the primary analysis, 17 received BFM chemotherapy, and 21 were treated according to the NOPHO (Table 2).

Post-induction measurable residual disease (MRD) assessment was available for all 38 patients. The majority of patients achieved CR after the end of the first course of induction (EOI) (25/38, 65.8%), of whom three (12.0%) were MRD-positive, suggesting that morphological remission did not uniformly reflect deep molecular response (Table 3). In contrast, all 13 patients with partial remission or refractory disease (34.2%) were MRD-positive. Overall, MRD positivity was detected in 16 of 38 patients (42.1%). These findings support the potential complementary value of MRD assessment alongside morphological response evaluation for post-induction risk stratification in pediatric AML. However, given the limited sample size, these observations should be interpreted as exploratory rather than conclusive.

Although induction response suggested favorable early treatment outcomes in a substantial proportion of patients, genomic risk stratification demonstrated that response-based assessment alone did not fully capture underlying disease risk.

Genomic profiling showed that only seven patients were classified as low-risk, whereas ten were assigned to the intermediate-risk group, and the majority (21/38, 55.3%) were classified as high-risk. The prognostic significance of MRD status after end of induction (EOI) was further illustrated by the clinical outcomes observed across risk subgroups. Among the 25 patients who achieved CR with MRD negativity after EOI, three patients nonetheless experienced adverse outcomes: one died due to treatment-related infectious complications, while two relapsed with fully refractory disease and subsequently died, underscoring that MRD negativity at EOI, although associated with favorable induction response, does not uniformly preclude disease recurrence or treatment-related mortality. These cases highlight the limitations of a single post-induction MRD assessment as a standalone prognostic tool and suggest that dynamic MRD monitoring beyond EOI may be necessary to capture evolving disease biology.

Among intermediate-risk patients, the two MRD-negative cases proceeded to allogeneic HSCT and remained alive at more than two years post-transplant, suggesting that MRD negativity in the context of intermediate genomic risk may identify a subgroup with favorable post-transplant outcomes; however, the very small numbers preclude any definitive conclusion.

In the high-risk group, 15 patients were MRD-negative after EOI, of whom 12 proceeded to allogeneic HSCT. Despite MRD negativity at the time of transplant, three patients died: one from severe infectious complications in the early post-transplant period, one from very early post-transplant relapse, and one from refractory disease prior to transplant. These observations suggest that, in high-risk genomic disease, MRD negativity after induction may be insufficient to overcome the adverse biological features driving treatment resistance and early relapse, and that additional therapeutic strategies—including post-transplant maintenance or novel targeted agents—may be required in this subgroup.

Taken together, these findings support the potential role of MRD status as a complementary prognostic factor in pediatric AML, particularly when interpreted in conjunction with genomic risk category and induction response.

This indicates that a large proportion of patients with an initially favorable clinical response were subsequently reclassified into biologically more adverse risk categories based on genomic findings. This discordance was particularly relevant for treatment consolidation. These findings suggest that genomic risk stratification provided clinically meaningful refinement beyond induction response alone, identifying patients with persistent high-risk disease biology despite achieving remission and thereby influencing post-remission treatment allocation. Genomic risk refined treatment allocation but did not significantly discriminate OS in this cohort (Table 4). Of the 7 low-risk patients, 6 achieved CR after induction (85.7%); of the 10 intermediate-risk patients, 8 achieved CR (80%); of the 21 high-risk patients, 11 achieved CR (52.4%). Regarding survival in non-transplanted patients: the remaining 14 patients did not proceed to transplant, either due to refractory disease (*n* = 10), early death during induction (*n* = 2), or low-risk genomic profile not meeting transplant criteria (*n* = 2—both low-risk patients in sustained CR). Among non-transplanted patients, crude 2-year OS was 21.4%, reflecting the predominance of refractory and high-risk disease in this subgroup.

**Figure 1:**
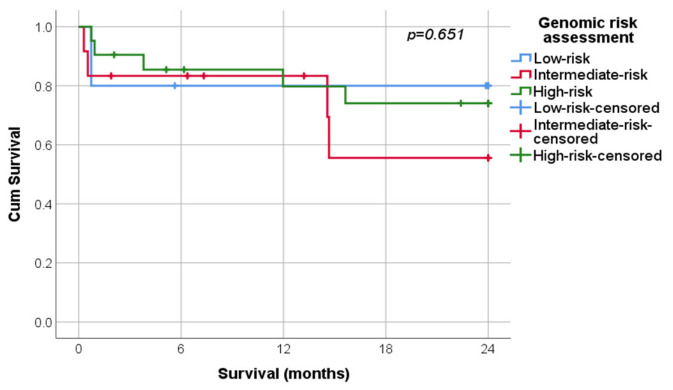
Kaplan–Meier overall survival according to genomic risk stratification.

A total of 24 patients underwent allogeneic HSCT based on genomic risk assessment (Figure 1). One patient, initially classified as low risk according to ELN 2022 criteria [25] (noting that BFM/NOPHO-based criteria were applied in parallel for transplant decision-making, as described in Section 4.2) with *RUNX1*–*RUNX1T1* fusion, underwent MUD allo-HSCT in a second complete remission following early relapse. This patient experienced very early post-transplant relapse and was treated with chemotherapy, azacitidine, and donor lymphocyte infusion (DLI), but remained refractory and subsequently died.

In the intermediate-risk group, nine patients underwent HSCT. Three patients harbored *KMT2A*-associated rearrangements, including two with *KMT2A::MLLT3* and one with *KMT2A::RARA*. Six patients underwent MUD allo-HSCT, of whom three received myeloablative conditioning (MAC) and three reduced-toxicity conditioning (RTC). Three patients underwent haploidentical HSCT with reduced-intensity conditioning (RIC). Two patients relapsed after transplantation and died, both with FLT3-positive disease, one of them receiving chemo + DLI with no benefit.

The high-risk transplanted subgroup comprised 14 patients and showed marked genomic heterogeneity (Table 5). Eleven patients underwent MUD allo-HSCT, two underwent haploidentical HSCT, and one received an MSD transplant. The patient harboring a *MECOM* rearrangement relapsed after transplantation and died. One patient carrying *FLT3*, *NPM1*, and *DNMT3A* alterations developed acute skin and gut graft-versus-host disease (GVHD) and chronic lung GVHD requiring topical and systemic corticosteroids, ruxolitinib, and photopheresis, with a subsequent fatal outcome.

Patients harboring *FLT3* alterations were assigned to the intermediate-risk group according to the ELN 2022 classification, which does not incorporate *FLT3* allelic ratio in its current risk framework [26]. The only patient with an *FLT3* alteration classified as high-risk also harbored concurrent *NPM1* and *DNMT3A* alterations and experienced an unfavorable outcome.

**Figure 2:**
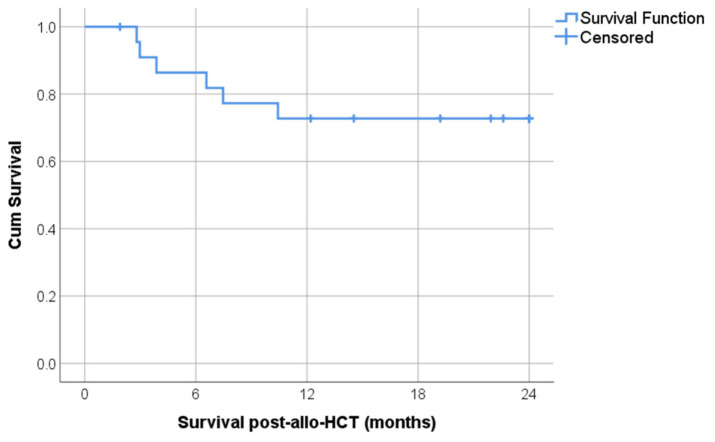
Kaplan–Meier post-transplant overall survival.

Post-transplant survival following allo-HSCT was favorable in this cohort (Figure 2). Overall survival remained above 80% during the early post-transplant period and stabilized at approximately 72.9% beyond 10 months after transplantation. Most post-transplant events occurred within the first year, after which the survival curve plateaued, suggesting durable disease control among surviving patients. At the last follow-up, 17/24 transplanted patients (70.8%) were alive, corresponding to a Kaplan–Meier-estimated 2-year post-transplant overall survival rate of 72.9%. These findings indicate that allo-HSCT provided sustained post-remission disease control in a substantial proportion of high-risk pediatric AML patients, although early post-transplant mortality and relapse remained clinically relevant.

Cytogenetic analysis was available for the majority of patients (see Table 6). A normal karyotype was observed in 50% of cases, while 18.4% exhibited a complex karyotype, and 10.5% had no evaluable metaphases. FISH analysis was negative in 63.1% of patients. Among detected abnormalities, deletion 7q (5.3%) and deletion 17p were the rarest (5.3%) at the same frequency.

**Figure 3:**
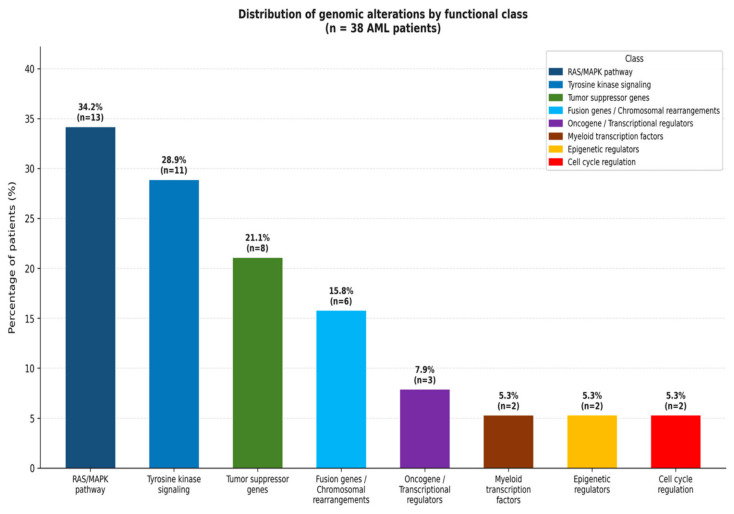
Frequency of recurrent genetic alterations in the total cohort. Created in BioRender. Ana, A. (2026), https://BioRender.com/5q177af, accessed on 7 June 2026.

Genomic alterations were grouped into major functional pathways according to their biological class (Figure 3). RAS/MAPK pathway alterations were the most frequent, identified in 13 patients (34.2%), followed by tyrosine kinase signaling alterations in 11 patients (28.9%). Fusion genes and chromosomal rearrangements were present in six patients (15.8%), and tumor suppressor gene alterations (eight patients, 21.1%). Oncogene/transcriptional regulator abnormalities were identified in three patients (7.9%), while myeloid transcription factor alterations, epigenetic regulator abnormalities, and cell cycle regulator alterations were each present in two patients (5.3%). Overall, this distribution underscores the marked molecular heterogeneity of the cohort. Given the limited sample size, these frequencies should be interpreted as descriptive rather than confirmatory.

**Figure 4:**
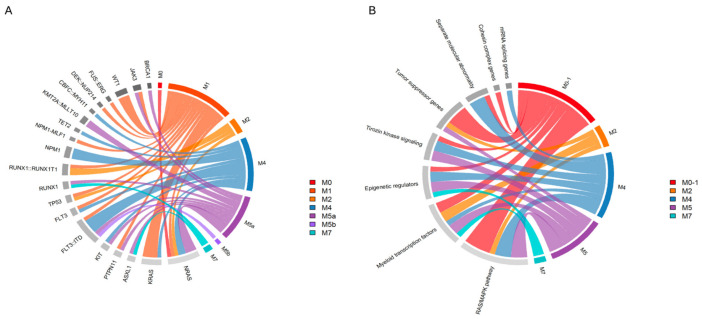
(**A**,**B**). Circos plots illustrating the distribution of genetic alterations across FAB subtypes in pediatric AML. Created in BioRender. Ana, A. (2026), https://BioRender.com/2z0mlaa, accessed on 7 June 2026.

The Circos plot (Figure 4A,B) illustrates the distribution of recurrent genetic alterations across FAB subtypes in pediatric AML. Each segment represents a FAB subtype, while connecting chords indicate the presence of specific genetic alterations grouped by functional pathways. The visualization demonstrates once again a heterogeneous distribution of mutations across all FAB categories, with no strict one-to-one correspondence between genetic alterations and morphological subtypes. Notably, alterations in the RAS/MAPK and tyrosine kinase signaling pathways are broadly distributed across multiple FAB subtypes, rather than being subtype-specific. In contrast, certain genetic alterations show preferential clustering within specific subtypes. For example, rearrangements involving core-binding factor genes and *KMT2A* are more frequently associated with monocytic and myelomonocytic phenotypes (M4/M5), reflecting known biological correlations in pediatric AML. Similarly, transcription factor alterations, including *RUNX1*, are observed across multiple FAB subtypes, underscoring their heterogeneous clinical impact. The extensive overlap of genetic alterations across FAB categories highlights the limitations of morphology-based classification and reinforces the importance of integrated genomic profiling. Overall, the Circos plot supports the concept that pediatric AML is driven by complex molecular networks that transcend traditional morphological boundaries.

**Figure 5:**
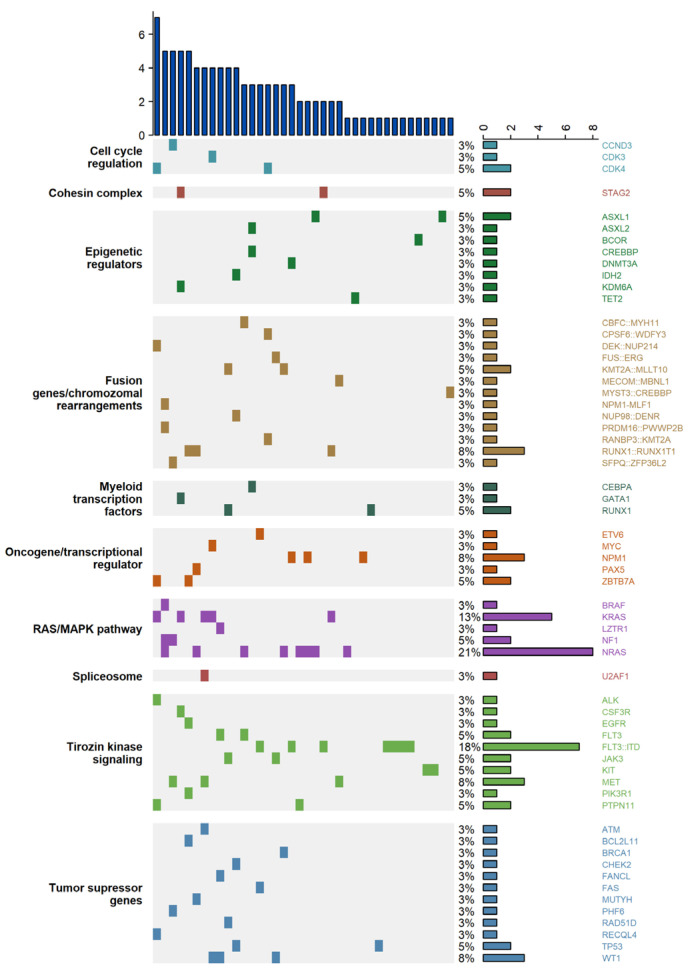
Heatmap of recurrent genetic alterations across functional pathways in pediatric acute myeloid leukemia.

The heatmap depicts the distribution of genomic alterations across individual patients, grouped by functional pathway class (Figure 5), and illustrates the marked interpatient heterogeneity of pediatric AML and the absence of a single dominant molecular lesion, supporting the need for comprehensive multi-gene profiling in this disease.

Table 7 summarizes the distribution of recurrent genomic alterations across the cohort, including their frequency, demographic characteristics, FAB subtype distribution, and crude EFS and OS. Among recurrent alterations, *NRAS* (*n* = 8), *KMT2A* rearrangements (*n* = 3), and *FLT3* mutations (*n* = 7) were the most frequently observed events. *NRAS* alterations were associated with favorable crude outcomes (EFS 100%; OS 100%), whereas *FLT3*-mutated cases showed inferior survival (EFS 8.6%; OS 13.6%). *RUNX1* alterations, including *RUNX1* fusion genes, were observed across morphologically heterogeneous subtypes and were associated with good survival. *TP53*-altered cases were rare (*n* = 2) and showed poor outcomes, although interpretation is limited by sample size. Overall, recurrent genomic alterations demonstrated again substantial heterogeneity across age, morphology, and survival.

Collectively, these findings demonstrate a highly heterogeneous mutational landscape spanning multiple biological pathways, with a predominance of signaling and transcriptional alterations.

Table 8 summarizes the results of univariable Cox regression analysis for EFS. Given the limited sample size and low number of events, all findings should be interpreted as exploratory observations rather than definitive prognostic conclusions, and the instability of Cox estimates in this context is acknowledged. Among the evaluated variables, high cytogenetic risk, high molecular risk, and *FLT3-ITD* showed a suggestive association with inferior EFS. High cytogenetic risk was associated with an approximately tenfold increased hazard of an event compared with favorable/intermediate cytogenetics (HR 10.091, 95% CI 1.299–78.398, *p* = 0.027); however, the wide confidence interval reflects the small number of events, and these data should be interpreted with caution. High molecular risk showed a similarly suggestive association with inferior EFS (HR 8.658, 95% CI 1.115–67.209, *p* = 0.039), though again the broad confidence interval limits the reliability of this estimate. *FLT3-ITD* was the most robustly associated variable, with a more than sevenfold increased hazard of an event (HR 7.208, 95% CI 2.135–24.342, *p* = 0.001), even after incorporation of *FLT3*-directed TKI therapy into frontline treatment for *FLT3*-positive patients; this finding, while consistent with published literature, requires confirmation in larger prospective cohorts. By contrast, age at diagnosis, *NRAS*, and *RUNX1* were not significantly associated with EFS, with confidence intervals spanning several orders of magnitude for the latter two variables, further illustrating the limitations of univariable regression in this small cohort.

No multivariable analysis was performed, because the number of events was insufficient for reliable model estimation. Given the low number of events and sparse representation of several molecular subgroups, these findings should be considered exploratory and interpreted with caution.

## 3. Discussion 

In this study, we provide a descriptive characterization of clinical, cytogenetic, and molecular features in a small single-center cohort of 38 pediatric AML patients, with exploratory observations regarding their potential impact on treatment response and survival outcomes. A notable finding was the high proportion of patients presenting with adverse disease characteristics, including a predominance of high-risk disease (55.3%) and substantial molecular heterogeneity. This distribution is consistent with previous reports emphasizing the biological and clinical complexity of pediatric AML [18]. Despite this, treatment outcomes following HSCT were encouraging in this selected group; it should be noted, however, that the 24 transplanted patients represent a biologically favorable subset—those who survived induction and achieved at least morphological remission—and survival estimates in this group are therefore subject to selection bias. With this important consideration in mind, 18 of 24 transplanted patients (75%) were in complete remission at last follow-up, with a Kaplan–Meier estimated 2-year post-transplant overall survival rate of 72.9%. In this exploratory analysis, both dynamic response to induction and genomic risk stratification appeared to provide clinically relevant prognostic information; no formal comparative analysis between the two approaches was performed, and no claim of superiority of one over the other can be made on the basis of these data. While genomic profiling frequently reclassified patients with an initially favorable clinical response into biologically higher-risk categories—with 55.3% ultimately assigned to high genomic risk despite an initial response rate of 73.7%—and informed post-remission treatment allocation, induction response showed the clearest separation in overall survival curves. However, given the small sample size and the retrospective single-center design, this observation should be regarded as hypothesis-generating rather than conclusive. Relapse remained a major cause of treatment failure, consistent with prior studies demonstrating that disease recurrence is the leading cause of treatment failure in pediatric AML, particularly in high-risk groups [16].

At the molecular level, our findings suggest the potential prognostic relevance of selected recurrent genomic alterations. *FLT3* mutations were associated with descriptively inferior survival (EFS 8.6%, OS 13.2%) despite uniform incorporation of sorafenib into frontline therapy, an observation consistent with their well-established role as adverse prognostic markers in AML [27,28]; however, the small subgroup size (*n* = 7) and wide confidence intervals preclude definitive conclusions. FLT3-ITD allelic ratio data were not available for all *FLT3*-positive patients, precluding its inclusion as a prognostic variable.

Similarly, *TP53*-altered cases showed descriptively poor outcomes (EFS 0%, OS 50%), consistent with prior reports linking *TP53* alterations to genomic instability, treatment resistance, and adverse prognosis in AML [15,29,30,31]; given that only two patients harbored *TP53* alterations in this cohort, no prognostic inference can be drawn beyond a purely descriptive level.

In contrast, selected genomic subgroups demonstrated descriptively more favorable outcomes. *KMT2A* alterations were identified in three patients in the intermediate-risk group, who underwent HSCT with subsequent complete remission and sustained complete donor chimerism. These findings are consistent with the known variability outcomes in *KMT2A*-rearranged AML [32], but given the small numbers, they should not be generalized beyond this cohort.

RAS pathway mutations (*NRAS* and *KRAS*) were among the most frequently observed alterations and were associated with descriptively intermediate to favorable survival (*NRAS*: EFS 100%, OS 100%; *KRAS*: EFS 75%, OS 75%). The prognostic significance of RAS mutations in AML remains heterogeneous in the literature and appears strongly influenced by co-occurring genomic lesions [33,34]; the apparent favorable association observed here may therefore reflect co-mutation context rather than an independent prognostic effect, and should be regarded as an exploratory observation. *NPM1* mutations (*n* = 3) were associated with descriptively more favorable outcomes (EFS 66.67%, OS 66.67%), consistent with reports of relatively favorable prognosis in *NPM1*-mutated AML in the absence of co-occurring high-risk mutations [35]; however, the subgroup was too small for any independent prognostic inference.

The heterogeneous genomic landscape of this cohort further underscores the biological complexity of pediatric AML and the need for refined molecular classification and risk-adapted therapeutic strategies. The integration of cytogenetic and molecular data aligns with current trends toward genomics-driven risk stratification in AML [36], though the present study can contribute only descriptive observations in this direction.

The 2-year overall survival rate of 69.9% and event-free survival rate of 65.9% observed in this cohort fall within the ranges reported by contemporary cooperative-group studies, where EFS rates of 45–63% and OS rates of 65–80% have been described [20]. This broad consistency supports the comparability of our findings with larger pediatric AML cohorts despite the single-center design. However, these figures should be interpreted in the context of the study’s limitations: the small cohort size (*n* = 38) and low number of events within molecular subgroups substantially limit statistical power and preclude definitive conclusions regarding the prognostic impact of most alterations. No formal power calculation was performed, and the study should be considered exploratory in its entirety, particularly with respect to subgroup analyses. The marked genomic heterogeneity of the cohort further limited the interpretability of low-frequency alterations and precluded robust multivariable modeling. Future prospective multicenter studies with larger sample sizes and standardized genomic profiling pipelines will be necessary to validate the exploratory associations described.

Post-transplant outcomes in this cohort reflected once again the biological heterogeneity of the transplanted population. Among the 24 patients who underwent allogeneic HSCT, clinically significant post-transplant events were observed across all risk groups. In the low-risk group, one patient with *RUNX1::RUNX1T1* relapsed very early after transplantation and died despite salvage therapy with chemotherapy, azacitidine, and DLI, illustrating that even genomically favorable disease can follow an aggressive clinical course following relapse. In the intermediate-risk group, two *FLT3*-positive patients relapsed post-transplant and died, one without benefit from chemo-DLI salvage, underscoring the persistent adverse impact of *FLT3*-positive disease even in the context of transplantation. In the high-risk group, the patient harboring a *MECOM::MBNL1* rearrangement experienced early post-transplant relapse with fatal outcome, consistent with the known poor prognosis of *MECOM*-rearranged AML [20]. One additional patient with concurrent *FLT3*, *NPM1*, and *DNMT3A* alterations developed severe acute and chronic GVHD refractory to multimodal immunosuppression—including systemic corticosteroids, ruxolitinib, and photopheresis—with subsequent fatal outcome in the absence of relapse, highlighting transplant-related mortality as a distinct cause of death beyond disease recurrence. Given the small numbers, these observations are purely descriptive and should not be generalized beyond this cohort.

The prognostic relevance of post-induction MRD status observed in this cohort is consistent with the established literature recognizing MRD as one of the strongest independent predictors of outcome in pediatric AML [37]. In our cohort, MRD positivity was strongly associated with suboptimal induction response, while MRD negativity after CR did not uniformly preclude adverse outcomes, particularly in the high-risk genomic subgroup. These findings suggest that MRD assessment provides prognostic information complementary to both morphological response and genomic risk stratification, and may help identify patients at highest risk of treatment failure who could benefit from treatment intensification or novel therapeutic approaches.

Additional methodological limitations include the use of a single pre-specified MRD threshold (<0.01%), with no formal testing of alternative cut-off points—an analysis that was not feasible given the retrospective design and limited sample size. Furthermore, the cohort reflects the inherent biological and treatment-related heterogeneity of pediatric AML, including the concurrent use of two induction protocols (AML-BFM and NOPHO) across the study period, which may independently influence survival outcomes and MRD kinetics and further limits the interpretability of subgroup comparisons. These limitations are inherent to retrospective single-center studies and reinforce the need for prospective multicenter designs with standardized protocols and pre-specified analytical frameworks.

## 4. Materials and Methods

### 4.1. Study Design and Patients

This retrospective single-center observational study was conducted at the Fundeni Clinical Institute in Bucharest and included pediatric patients (≤18 years) diagnosed with AML treated between 2020 and 2025. Patients with mixed-phenotype acute leukemia (MPAL) were excluded, as MPAL represents a biologically distinct entity that requires separate analysis; we also excluded AML associated with Down syndrome and AML M3 (FAB). Diagnosis was established based on bone marrow morphological examination, flow cytometry immunophenotyping, cytogenetic analysis, FISH, molecular biology testing, and NGS. Patients received chemotherapy and, depending on risk classification, underwent allogeneic-HSCT. Patients with high risk of relapse received additional post-transplant therapy, including DLI and azacitidine therapy. Mixed-donor chimerism (MDC) was defined as the presence of 5–95% donor cells determined by short tandem repeat (STR) analysis, according to EBMT guidelines [38]. Measurable residual disease positivity was defined as levels exceeding 0.01% leukemic blasts in bone marrow samples assessed by flow cytometry.

Immunophenotypic analysis was performed by flow cytometry using the BD FACSLyric™ system (Becton Dickinson, Franklin Lakes, NJ, USA), with data interpreted using Infinicyt™ software version 2.0 (Cytognos, Salamanca, Spain) at a sensitivity of 10^−4^. Conventional cytogenetic analysis was conducted using GTG banding, and FISH was performed in accordance with protocol-specific recommendations. Recurrent fusion transcripts were evaluated by multiplex RT-PCR, and targeted mutational profiling was performed using the Illumina TruSight™ Oncology 500 platform (Illumina, San Diego, CA, USA). Somatic variant calling was performed with a minimum variant allele frequency (VAF) threshold of 5%, consistent with the validated analytical sensitivity of the TruSight Oncology 500 platform. Variants with VAF ≥ 45% in the diagnostic sample were considered potentially germline and underwent additional assessment using matched non-tumor DNA (peripheral blood in remission or buccal swab) where available. Bioinformatic processing was performed using the Illumina DRAGEN pipeline with the Isaac Variant Caller (reference genome GRCh38). Fusion gene calls were generated by the RNA-seq module and retained after internal bioinformatic filtering and manual review; however, two events (*SFPQ::ZFP36L2* and *CPSF6::WDFY*) could not be validated by orthogonal methods (RT-PCR, Sanger sequencing, or FISH) due to limited remaining material, and should therefore be considered putative findings. Variant classification was performed in accordance with the joint consensus recommendations of the Association for Molecular Pathology (AMP), the American Society of Clinical Oncology (ASCO), and the College of American Pathologists (CAP). According to these guidelines, genetic variants are stratified into tiers reflecting their clinical significance, ranging from Tier I (strong clinical significance) to Tier IV (benign or likely benign variants). In the context of acute myeloid leukemia (AML), Tier I variants include alterations with established clinical relevance, either as prognostic markers or as targets for risk-adapted therapeutic strategies. Tier IA variants are supported by Level A evidence, including established clinical guidelines and well-defined roles in AML management, while Tier IB variants are supported by Level B evidence derived from clinical studies or expert consensus. Tier II variants (including Tier IIC) represent alterations with potential clinical significance, supported by emerging or limited evidence, which may inform disease biology or therapeutic considerations. Variants classified as Tier III (variants of uncertain significance) and Tier IV (benign or likely benign) were not included in the present analysis. Accordingly, only variants categorized as Tier IA, IB, and IIC were considered for downstream analyses.

### 4.2. First-Line Therapy

Patients were treated according to the BFM protocol (cytarabine 100 mg/m^2^/d, day 1, 2; cytarabine 100 mg/m^2^ every 12 h, days 3–8; idarubicin 12 mg/m^2^/day, days 3, 5, 7; etoposide 150 mg/m^2^/day, days 6, 7, 8) between 2020 and 2022 and the NOPHO protocol (cytarabine 200 mg/m^2^ 12 h, days 6–12; mitoxantrone 5 mg/m^2^, days 6–10; etoposide 150 mg/m^2^, days 1–5) from 2022 onward.

All patients with *FLT3*-altered AML received the FLT3-directed tyrosine kinase inhibitor (TKI) sorafenib in combination with frontline chemotherapy. Risk stratification was based on response after the first induction cycle (day 22) and classified as follows: CR, defined as <5% leukemic blasts in the bone marrow at the end of induction, absence of blasts in peripheral blood (PB), and no extramedullary disease; partial remission (PR), defined as 5–25% leukemic blasts in the bone marrow, absence of blasts in PB, and no extramedullary disease; and refractory disease, defined as failure to achieve a response after induction. Genetic risk assessment was based on the underlying genomic profile and was considered independently of blast percentage after initial therapy, according to the ELN 2022 classification system [25]. Although ELN 2022 [25] was originally developed for adult AML, it was applied here because no universally validated pediatric-specific NGS-based risk framework was available at the time of the study. ELN 2022 [25] provides a structured classification incorporating molecularly defined markers (*FLT3-ITD*, *NPM1*, *CEBPA*, *TP53*, *ASXL1*, *RUNX1*) that are clinically relevant in pediatric AML and are routinely assessed in standard diagnostic workups. Its limitations in the pediatric context are acknowledged: notably, several age-specific high-risk entities prevalent in children—including *NUP98::NSD1*, *NUP98::KDM5A*, and certain *KMT2A* fusion partners—are not adequately captured by ELN 2022 [25] risk categories. Pediatric-specific risk stratification criteria (BFM/NOPHO protocol-based) were used in parallel for transplant decision-making, as described in Section 4.3. The use of risk stratification here should therefore be considered a structured reference framework rather than a definitive pediatric risk tool.

### 4.3. Transplant in CR1

Patients who underwent transplantation were classified as intermediate- or high-risk (with one patient initially in standard risk, but with relapse and CR2 at the time of HSCT) or harbored genomic alterations associated with adverse prognosis. According to the AML-BFM/SIOPE standard clinical practice recommendations, patients were considered eligible for allogeneic HSCT in first complete remission if classified as high-risk on the basis of inadequate response to induction therapy and/or adverse genetic features, including complex karyotype, del(5q), abnormalities of 3q, monosomy 5 or 7, and *FLT3-ITD* in combination with *WT1* alteration, provided that complete remission or at least morphologic leukemia clearance was achieved. According to the NOPHO-DBH AML 2012 protocol, indications for allogeneic HSCT in first complete remission included: poor response to induction therapy and/or persistent measurable residual disease (MRD), *FLT3-ITD*-positive AML without concomitant *NPM1* mutation, and selected very-high-risk cytogenetic abnormalities in the setting of suboptimal induction response. Donor sources included matched sibling donors (MSD), matched unrelated donors (MUD), and haploidentical donors. Conditioning regimens were categorized as myeloablative conditioning (MAC), reduced-toxicity conditioning (RTC), or reduced-intensity conditioning (RIC) as previously described by our group [39]. MAC consisted primarily of busulfan–cyclophosphamide-based regimens with or without melphalan. RTC included busulfan-based combinations with fludarabine/thiotepa or clofarabine, as well as reduced-toxicity regimens such as thiotepa–treosulfan–fludarabine. GVHD prophylaxis was adapted according to donor type: MSD recipients received a calcineurin inhibitor (CNI) with short-course methotrexate; MUD recipients received either antithymocyte globulin (ATG)-based prophylaxis or tacrolimus, mycophenolate mofetil, and post-transplant cyclophosphamide (PT/Cy); and haploidentical recipients uniformly received PT/Cy-based prophylaxis. Peripheral blood stem cells (PBSC) were the predominant graft source.

### 4.4. Post-Transplant Management

Measurable residual disease (MRD) and chimerism assessments were performed at 1 month and subsequently at 3, 6, 9, and 12 months post-transplantation, as well as whenever clinically indicated. Azacitidine and DLI were administered as preemptive or prophylactic therapy (5 patients) in cases of MDC or MRD positivity, or in high-risk patients, and therapeutically (2 patients) in cases of relapse, as previously described by our group [40]. All 7 patients received azacitidine at a dose of 75 mg/m^2^/day for 7 consecutive days every 4 weeks before DLI, except for those who received chemotherapy. DLI was administered in escalating doses ranging from 1 × 10^5^/kg to 1 × 10^7^/kg CD3^+^ cells. In patients who relapsed, additional treatment strategies included salvage chemotherapy regimens such as FLAG, GLAG-M, or DCAG.

### 4.5. Outcome and Follow-Up

The primary outcomes of the study were 2-year OS, EFS, and the achievement of complete donor chimerism for patients who underwent HSCT. Overall survival was defined as the time from diagnosis to death from any cause or the last follow-up. EFS was defined as the time from diagnosis to the first occurrence of an adverse event, including failure to achieve complete remission, disease relapse, or death from any cause. Patients without events were censored at the time of last follow-up. Complete donor chimerism was defined as the presence of 95–100% donor-derived hematopoiesis. Secondary outcomes included the incidence of GVHD and leukemia relapse. Acute and chronic GVHD were evaluated according to standard clinical criteria [41]. Relapse was defined as the reappearance of leukemic blasts in the bone marrow or peripheral blood, or the occurrence of extramedullary disease. Follow-up time was calculated from the date of diagnosis until death or the last recorded clinical evaluation.

The present study shares the same institutional setting as two previously published analyses from our group and, given the overlapping study periods, a partial overlap in patient population cannot be excluded. Bica et al. (Frontiers in Pharmacology, 2025, [40]) reported on 16 pediatric AML patients who received donor lymphocyte infusion combined with azacitidine following allogeneic HSCT between 2016 and 2024, with the primary aim of evaluating the safety and efficacy of this post-transplant intervention. Marcu et al. (Frontiers in Oncology, 2025, [39]) reported on 59 pediatric patients with myeloid malignancies who underwent allogeneic HSCT between 2015 and 2024, with the primary aim of comparing outcomes across three conditioning regimen intensities (MAC, RTC, and RIC). The present manuscript reports on 38 consecutive pediatric AML patients diagnosed and treated between 2020 and 2025, with the primary aim of characterizing the genomic landscape by next-generation sequencing and evaluating its integrated relationship with induction response kinetics and transplantation outcomes—a research question not addressed in either prior publication. The primary endpoints, analytical frameworks, and scientific hypotheses of the three studies are therefore distinct. No previously published data, results, tables, or figures have been reproduced in the present manuscript. The post-transplant interventions described in Section 4.4 include reference to the prior publications solely to provide methodological context and avoid redundant description of procedures already reported in detail elsewhere.

### 4.6. Statistical Analysis

Statistical analysis was performed using IBM SPSS Statistics version 25 (IBM Corp., Armonk, NY, USA) and R version 4.5.0 (R Foundation for Statistical Computing, Vienna, Austria) using RStudio 2026.05.1 (Posit Software, PBC, Boston, MA, USA).

Categorical variables were reported as numbers and percentages.

The Shapiro–Wilk test was used to assess the normality of the continuous variables. All continuous variables showed a non-normal distribution and were reported as medians and interquartile range (IQR).

Survival analysis was performed using the Kaplan–Meier method, and the differences between groups were evaluated using the log-rank test.

Hazard ratios with 95% confidence intervals were calculated using univariate Cox proportional hazard regression. Multivariate analysis was not feasible due to the limited number of events in subgroups.

The heatmap was generated using the ComplexHeatmap package, and the circular chord diagram was created using the circlize package.

AI-assisted tools were used for language editing and spelling correction. BioRender was used for figure design, and ChatGPT 5.5 (OpenAI) was used for minor text editing. All outputs were reviewed and verified by the authors.

No formal a priori sample size calculation was performed, because this was a retrospective single-center observational study including all consecutively diagnosed pediatric AML cases during the study period. Accordingly, the sample size was determined by case availability rather than statistical design, and all analyses should be considered exploratory.

### 4.7. Ethics

This observational study was conducted and reported in accordance with the STROBE (Strengthening the Reporting of Observational Studies in Epidemiology) guidelines and received approval from the Ethics Committee of the Fundeni Clinical Institute (approval no. 36105/29.08.2025). The study was non-interventional and retrospective in design; no study-specific diagnostic procedures, therapeutic interventions, or treatment modifications were introduced, and all patients were managed according to institutional standard-of-care protocols. Written informed consent was obtained from parents or legal guardians, including consent for the use of anonymized clinical and molecular data for research purposes, in accordance with the Declaration of Helsinki.

## 5. Conclusions

In conclusion, this exploratory single-center study suggests the potential complementary value of genomic profiling and early treatment response assessment in pediatric AML, while acknowledging that the limited sample size prohibits definitive prognostic conclusions. Integrated genomic assessment appeared to refine biologic risk stratification and inform post-remission therapeutic decision-making, particularly HSCT allocation; however, these observations should be interpreted as hypothesis-generating rather than confirmatory. In this cohort, both dynamic response to induction and genomic risk stratification showed descriptive association with survival outcomes, an observation consistent with published literature but requiring formal prospective validation, including comparative model performance analyses, in larger cohorts. It should be noted that survival estimates in the transplanted subgroup are subject to selection bias, as transplanted patients represent those who survived induction and achieved at least morphological remission. Although the 2-year OS rate of 69.9% and EFS rate of 65.9% observed in this cohort are broadly comparable to contemporary pediatric AML cohorts, relapse remained a major barrier to cure [42]. Future efforts should focus on prospective multicenter studies integrating genomic risk, dynamic response assessment, MRD monitoring, and targeted therapeutic strategies to improve long-term outcomes in pediatric AML.

## Figures and Tables

**Table 1:** Patients’ characteristics.

Indicators	No.	Percentage
Gender		
Male	23	60.5%
Female	15	39.5%
Age at diagnosis		
<1	3	7.9%
1–9 y	17	44.7%
≥10 y	18	47.4%
WBC (count × 10^9^/L)		
0–50	22	57.9%
50–100	5	13.2%
≥100	11	28.9%
FAB classification		
M0	1	2.6%
M1	11	28.9%
M2	2	5.3%
M4	10	26.3%
M5	12	31.6%
M6	0	
M7	2	5.3%
FU 2 years after HSCT (24 patients)		
CR	18	75%
EFS	9	37.5%
OS		72.9%
FU 2 years of the cohort		
EFS *	25	65.9%
2 y OS *		69.9%
Alive at last follow-up	25	65.9%

WBC—white blood cells; FAB—French-American-British; HSCT—hematopoietic stem cell transplant; FU—follow up; CR—complete remission; EFS—event-free survival; OS—overall survival. * Kaplan–Meier estimate at 24 months; values may differ from crude proportions because censoring is accounted for.

**Table 2:** Induction response by protocol.

Induction Response	CR	PR	Refractory Disease
AML-BFM *n* = 17	*n* = 11 CR 64.7%	*n* = 0 0	*n* = 6 Refractory 35.3%
NOPHO *n* = 21	*n* = 14 CR 66.6%	*n* = 3 PR 14.3%	*n* = 4 Refractory 19%
Overall treatment response	*n* = 25 CR (65.8%)	*n* = 3 PR (7.9%)	*n* = 10 Refractory (26.3%)

CR—complete remission; PR—partial remission.

**Table 3:** Measurable residual disease (MRD) positivity according to induction response after end of induction (EOI).

Response After EOI	No. of Patients	MRD Positivity
*CR*	25 (65.8%)	3 (12%)
*PR/Refractory*	13 (34.2%)	13 (100%)
*Total*	38 (100%)	16 (42.1%)

EOI—end of induction; MRD—measurable residual disease.

**Table 4:** Genomic risk stratification and HSCT allocation.

Genomic Risk Assessment	Low	Intermediate	High
Number of patients	7 (18.4%)	10 (26.3%)	21 (55.3%)
HSCT (*n* = 24)	1 (2nd CR)	9	14
Alive at last follow-up	0/1	6/9 (66.6%)	11/14 (78.6%)
2-year OS *	NA	66.7%	76.2%

* Kaplan–Meier estimate at 24 months.

**Table 5:** Genomic landscape in high-risk patients undergoing HSCT.

Patients	Genomic Lesion	Donor	Conditioning	Post-HSCT Relapse	2 y Status
*n* = 1	*KMT2A* + complex karyotype	MSD	RTC	No	Alive
*n* = 2	*NPM*1 + complex karyotype	MUD Haplo	RTC RIC	No No	Alive Alive
*n* = 2	Secondary AML + complex karyotype	Haplo MUD	RIC MAC	No No	Alive Alive
*n* = 1	*DEK::NUP214*	MUD	RTC	No	Alive
*n* = 1	Monosomy 11	MUD	MAC	No	Alive
*n* = 1	*KMT2A* + *MECOM::MBNL1*	MUD	RTC	Yes	Dead
*n* = 1	*FLT3* + *NPM1* + *DNMT3A*	MUD	RTC	No	Dead
*n* = 1	*RUNX1* somatic + KMT2A	MUD	RTC	No	Alive
*n* = 1	*KMT2A* rearrangements	MUD	MAC	No	Alive
*n* = 1	*WT1*	MUD	MAC	No	Alive
*n* = 1	*TP53*	MUD	RTC	No	Alive
*n* = 1	*PTPN11*	MUD	RTC	No	Alive

MUD—matched unrelated donor; MSD—matched sibling donor; MAC—myeloablative conditioning; RTC—reduced-toxicity conditioning; RIC—reduced-intensity conditioning.

**Table 6:** Cytogenetic landscape.

Genetic Mutations	No.	Percentage
No evaluable metaphases	4	10.5%
Normal karyotype	19	50%
Complex karyotype	7	18.4%
Chromosomal abnormality	11	28.9%
Trisomy 8	3	7.9%
Del 7q	2	5.3%
Del 17p	2	5.3%
FISH negative	24	63.1%

FISH—fluorescence in situ hybridization.

**Table 7:** Association between genomic alterations and clinical outcome.

Genomic Alteration	*n*	Median Age (Years)	Sex (M/F)	EFS (2 y)	OS (2 y)
**Tirozin kinase signaling**					
*JAK3*	2	8.84	0/2	100%	100%
*PTPN11*	2	12.18	0/2	100%	100%
*FLT3-ITD*	7	10.04	7/0	8.6%	13.16%
**RAS/MAPK Pathway**					
*KRAS*	5	7.60	1/4	75%	75%
*NRAS*	8	13.99	3/5	100%	100%
**Myeloid transcription factors**					
*RUNX1*	2	5.98	2/0	100%	100%
**Fusion genes/chromosomal rearrangements**					
*RUNX1 fusion genes*	3	9.10	1/3	100%	100%
*KMT2A rearrangement*	3	5.25	1/2	100%	100%
**Epigenetic regulators**					
*ASXL1*	2	0.54	1/1	50%	50%
**Tumor suppressor genes**					
*WT1*	3	10.42	1/2	100%	100%
*NPM1*	3	10.47	3/0	66.67%	66.67%
*TP53*	2	14.07	2/0	0%	50%
**Cell cycle regulation**					
*CDK4*	2	6.23	1/1	100%	100%
*Others*	25	9.10	17/14	69.54%	69.54%

EFS—event-free survival; OS—overall survival; Others (≤2 patients per alteration): *RAS/MAPK* pathway—*NF1*, *BRAF*, *CBL*, *LZTR1*. Myeloid transcription factors—*GATA1*, *CEBPA*. Epigenetic regulators—*TET2*, *ASXL2*, *KDM6A*, *BCOR*, *IDH2*. Tyrosine kinase signaling—*KIT*, *CSF3R*, *EGFR*, *PIK3R1*, *MET*, *FLT3*. Tumor suppressor genes—*ATM*, *CHEK2*, *FANCL*, *MUTYH*, *FAS*, *BCL2L11*, *PHF6*, *BRCA1*, *RAD51D*, *RECQL4*. Candidate fusion events/chromosomal rearrangements—*CPSF6::WDFY†*, *PRDM16::PWWP2B*, *MECOM::MBNL1*, *SFPQ::ZFP36L2†*, *ZNF384::TAF15*, *DEK::NUP214*, *FUS::ERG*, *MYST3::CREBBP*, *NPM1::MLF1*, *NUP98::DENR*, *CBFB::MYH11*. Cohesin-complex-associated genes—*STAG2*. Spliceosome—*U2AF1*. Cell cycle regulation—*CDK3*, *CCND3*. Oncogenes/transcriptional regulators—*MYC*, *ETV6*, *PAX5*, *ZBTB7A.* FLT3-ITD allelic ratio was not available for all cases and was therefore not reported. † *CPSF6::WDFY* and *SFPQ::ZFP36L2* are considered putative/exploratory findings; orthogonal validation (RT-PCR, Sanger sequencing, or FISH) was not available for these cases due to limited remaining material. *SFPQ::ZFP36L2* has been previously described in the context of chromothripsis in T-ALL rather than AML; its biological significance in this context requires further investigation.

**Table 8:** Univariable Cox regression analysis for EFS.

Variable	Hazard Ratio	95% CI	*p* Value
Age at diagnosis	0.920	0.820–1.032	0.156
Cytogenetic risk			
Favorable–Intermediate	Reference		
High	10.091	1.299–78.398	0.027
Molecular risk			
Favorable–Intermediate	Reference		
High	8.658	1.115–67.209	0.039
NGS *			
*NRAS*	0.031	0.000–7.545	0.215
*FLT3-ITD*	7.208	2.135–24.342	0.001
*RUNX1*	0.045	0.000–967.704	0.541

NGS *—reference category corresponds to the absence of respective mutation.

## Data Availability

The data presented in this study are available on request from the corresponding author due to ethical reasons.

## Abbreviations

The following abbreviations are used in this manuscript:

AML	Acute myeloid leukemia
MPAL	Mixed-phenotype acute leukemia
NGS	Next-generation sequencing
WBC	White blood cell
FAB	French-American-British
LR	Low risk
IR	Intermediate risk
HR	High risk
HSCT	Hematopoietic stem cell transplantation
FU	Follow-up
CR	Complete remission
AZA	Azacitidine
DLI	Donor lymphocyte infusion
EFS	Event-free survival
OS	Overall survival
PR	Partial remission
MAC	Myeloablative conditioning
RTC	Reduced-toxicity conditioning
RIC	Reduced-intensity conditioning
ELN	European Leukemia Net
allo-HSCT	Allogeneic stem cell transplantation
MUD	Matched unrelated donor
MSD	Matched sibling donor
GVHD	Graft-versus-host disease
FISH	Fluorescence in situ hybridization
NA	Not applicable
AMP	Association for Molecular Pathology
ASCO	American Society of Clinical Oncology
CAP	College of American Pathologists
TKI	Tyrosine kinase inhibitor
PB	Peripheral blood
CI	Calcineurin inhibitor
MRD	Measurable residual disease
MDC	Mixed-donor chimerism
STR	Short tandem repeat
VAF	Variant allele frequency
PBSC	Peripheral blood stem cells
PT/Cy	Post-transplant cyclophosphamide
ATG	Anti-thymocyte globulin
IQR	Interquartile range
STROBE	Strengthening the Reporting of Observational Studies in Epidemiology
EOI	End of induction
